# Evidence-Based Physical Therapy Management for Chronic Ankle Instability in Young Females: A Systematic Review

**DOI:** 10.7759/cureus.110739

**Published:** 2026-06-12

**Authors:** Vaibhav V Shinde, Siddhi P Patrekar, Sandeep Shinde, Sakshi S Desai

**Affiliations:** 1 Department of Musculoskeletal Sciences, Krishna College of Physiotherapy, Krishna Vishwa Vidyapeeth (Deemed to be University), Karad, IND; 2 Department of Orthopedic Manual Therapy, Krishna College of Physiotherapy, Krishna Vishwa Vidyapeeth (Deemed to be University), Karad, IND

**Keywords:** balance training, chronic ankle instability, lateral ankle sprain, neuromuscular rehabilitation, physiotherapy, proprioception

## Abstract

Chronic ankle instability (CAI) is a prevalent and debilitating musculoskeletal condition, particularly among young active females, resulting from recurrent lateral ankle sprains and subsequent neuromuscular deficits. Despite its high incidence in this demographic, evidence-based physiotherapy protocols tailored specifically to young female populations remain understudied. The primary aim of this review was to analyze evidence-based physical therapy interventions for CAI in young females, synthesize current findings on therapeutic efficacy, and identify gaps that warrant further investigation. A thorough online search for papers published between 2014 and 2026 was conducted using the Preferred Reporting Items for Systematic Reviews and Meta-Analyses (PRISMA) 2020 methodology across PubMed, Google Scholar, Scopus, Excerpta Medica Database (EMBASE), and the Cochrane Library. Thirteen studies satisfied the requirements for inclusion. The Risk of Bias-2 (RoB-2) tool for randomized controlled trials (RCTs) and the AXIS tool (appraisal tool for cross-sectional studies) were used to evaluate methodological quality. The selected research frequently indicated that structured physiotherapy encompassing balance training, neuromuscular re-education, peroneal strengthening, whole-body vibration, joint mobilization, proprioceptive neuromuscular facilitation (PNF) stretching, core stabilization, and multimodal rehabilitation significantly improves dynamic balance, proprioception, functional performance, and self-reported function in young females with CAI. Multimodal physiotherapy targeting neuromuscular and sensorimotor deficits is highly effective for CAI management in young females. Early identification, individualized programming, and integration of core and proximal strengthening into rehabilitation protocols are essential for optimizing outcomes and reducing reinjury risk.

## Introduction and background

Lateral ankle sprains account for a significant percentage of all sports-related injuries globally, making them one of the most common musculoskeletal conditions in both athletic and general populations. In India, 73% of athletes have chronic ankle instability (CAI), and 59% of them have experienced ankle sprains in the past [1]. Prospective epidemiological research shows that lateral ankle sprains influence people of all ages and activity levels, frequently leading to significant time lost from sport and physical activity. The elevated risk and frequency of recurrence of these injuries have been extensively documented [2].

A particularly important yet underexplored dimension of ankle sprain epidemiology is the influence of biological sex on injury patterns, recovery trajectories, and long-term outcomes. Recent data indicate that women may demonstrate distinct anatomical, hormonal, and biomechanical characteristics that predispose them to lateral ankle ligament injury and subsequently influence surgical and conservative treatment outcomes. Differences in ligamentous laxity, neuromuscular control strategies, and hormonal fluctuations are proposed as contributing factors to sex-specific vulnerability and recovery profiles following lateral ankle injury [3].

When lateral ankle sprains fail to resolve adequately, a substantial proportion of individuals, estimated at between 40% and 70%, develop CAI, a condition characterized by persistent feelings of giving way, recurrent sprain episodes, and residual sensations of instability lasting beyond 12 months from the initial injury [4]. The patho-mechanics of CAI are complex and multifactorial, encompassing both mechanical and perceived components. Established risk factors include deficits in proprioception, impaired postural control, reduced peroneal muscle reaction time, and altered movement patterns, all of which interact to perpetuate the cycle of instability and reinjury [5].

Females may contribute to increased susceptibility to CAI due to a wider pelvis, which increases the Q-angle and shifts mechanical load laterally onto the ankle joint, placing the lateral ligaments under greater chronic stress. Hormonally, estrogen and relaxin reduce ligamentous stiffness and collagen tensile strength, meaning the anterior talofibular ligament (ATFL) and calcaneofibular ligament (CFL) are inherently laxer and more vulnerable to giving way under load, which acts as one of the risk factors [5]. Biomechanically, women tend to land with greater ankle inversion and less knee flexion during dynamic tasks, and their peroneal muscles fire later in response to sudden perturbation compared to males, a delayed neuromuscular reaction that leaves the lateral ankle unprotected at the critical moment of contact. Combined with generally lower peroneal muscle strength relative to body mass, these anatomical and hormonal realities create a compounding vulnerability that makes the female ankle structurally less equipped to resist the inversion forces that drive instability.

From a physical therapy perspective, the management of CAI has changed significantly in recent decades, with an increasing amount of data demonstrating the effectiveness of targeted neuromuscular and proprioceptive rehabilitation strategies. Among these, proprioceptive neuromuscular facilitation (PNF) techniques have received increased attention, as recent systematic reviews and meta-analyses demonstrate significant improvements in balance, functional performance, and perceived instability in individuals with CAI [6]. Nonetheless, the broader evidence base encompasses a wide range of interventions, including balance training, resistance exercise, manual therapy, taping, and multimodal rehabilitation programs, each with varying levels of methodological rigor and clinical applicability.

Despite the breadth of existing literature, there remains a notable gap in evidence specifically examining physical therapy outcomes in young female populations with CAI. CAI is not merely an acute injury sequela but a long-term situation that has major implications for joint health, physical function, and quality of life if inadequately managed [7]. Young females constitute a clinically distinct subgroup whose specific physiological traits may influence the progression of CAI and the effectiveness of rehabilitation; however, they are infrequently the focus of targeted systematic evaluation.

**Figure 1:**
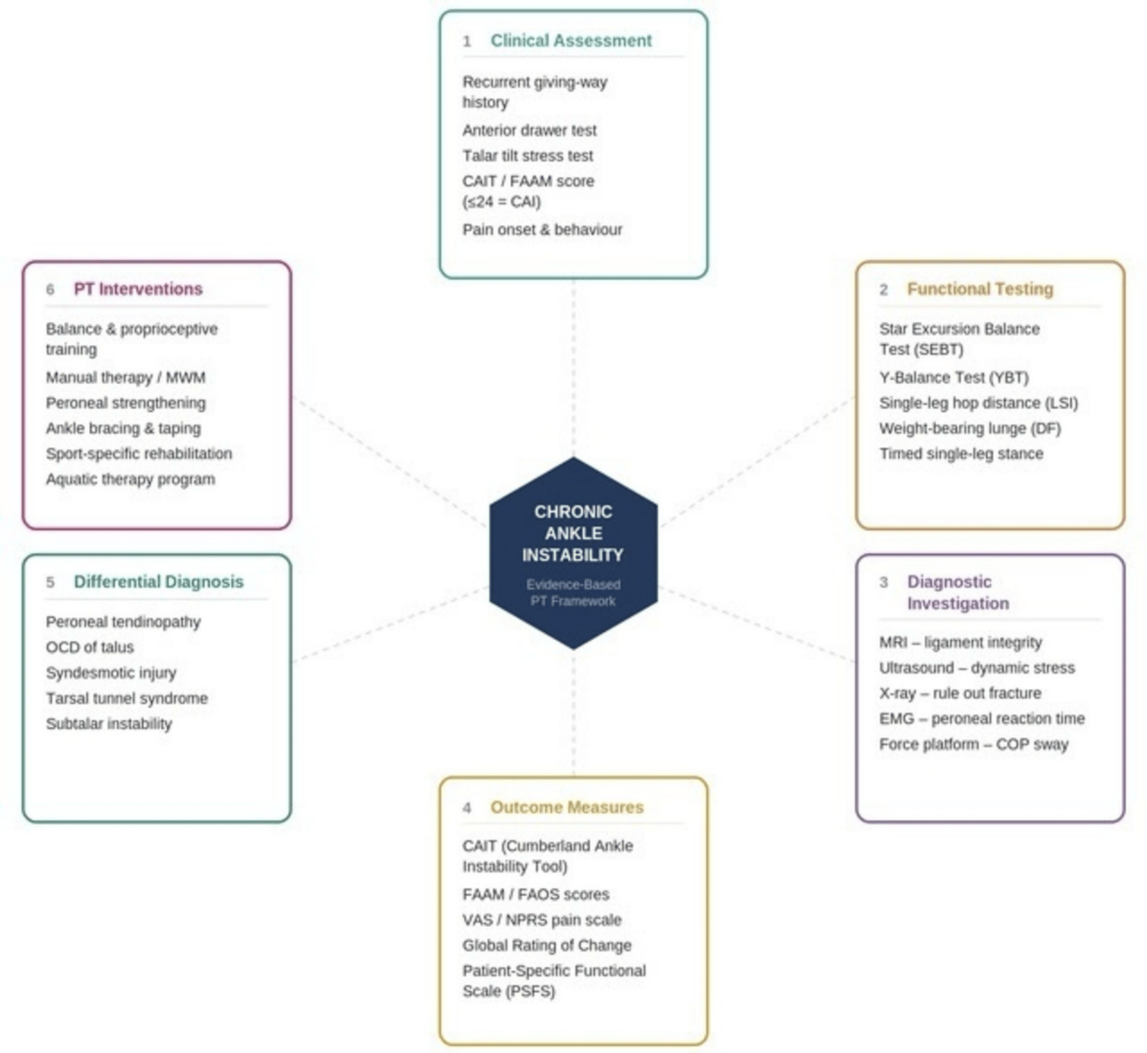
Comprehensive clinical framework for CAI CAI: chronic ankle instability; CAIT: Cumberland Ankle Instability Tool; FAAM: Foot and Ankle Ability Measure; SEBT: Star Excursion Balance Test; YBT: Y-Balance Test; LSI: Limb Symmetry Index; DF: dorsiflexion; MRI: magnetic resonance imaging; EMG: electromyography; COP: centre of pressure; VAS: Visual Analogue Scale; NPRS: Numeric Pain Rating Scale; FAOS: Foot and Ankle Outcome Score; PSFS: Patient-Specific Functional Scale; MWM: mobilisation with movement; PT: physical therapy The structure, design, and formatting of the figure were produced using Microsoft PowerPoint (Microsoft Corporation, Redmond, Washington, United States).

Also, the comprehensive clinical framework of CAI mainly has the outline of six key domains arranged in a manner such as clinical assessment, functional testing, diagnostic investigations, outcome measures, differential diagnosis, and physiotherapy management (Figure 1).

Evidence-based physical therapy strategies for managing CAI in young female populations are thoroughly evaluated and synthesized in this systematic review. This aims to direct clinical practice and identify areas of interest for further study.

## Review

Methodology

Study design

This systematic review was conducted and reported in accordance with the Preferred Reporting Items for Systematic Reviews and Meta-Analyses (PRISMA) 2020 framework to ensure transparency, reproducibility, and methodological rigor [8]. The revised Cochrane Risk of Bias (RoB) tool was used to assess RoB in randomized controlled trials (RCTs) [9]. For cross-sectional studies, an appraisal tool was used to determine risk [10].

Search Strategy

A thorough electronic database search spanning publications from 2014 to 2026 was carried out using PubMed, Google Scholar, Scopus, Excerpta Medica Database (EMBASE), and the Cochrane Library. The following MeSH terms and freely accessible keywords were used: "lateral ankle sprain," "chronic ankle instability," "functional ankle instability," "young females," "women athletes," "physical therapy," "physiotherapy," "balance training," "neuromuscular rehabilitation," "proprioception," "peroneal strengthening," "joint mobilization," "whole body vibration," "PNF," "core stability," and "ankle rehabilitation." To maximize search sensitivity, Boolean operators (AND, OR) were used. Manual screening was also done on the reference lists of the included papers and pertinent systematic reviews.

Inclusion Criteria

Research was included if it investigated participants diagnosed with CAI or functional ankle instability using validated criteria (e.g., International Ankle Consortium guidelines [11], CAIT score ≤ 4); included young female participants (predominantly aged 18-40 years); had severe ankle pain ≤ 12 months; evaluated physiotherapy-based interventions (exercise, manual therapy, electrophysical agents, multimodal programs); were RCTs, prospective/retrospective observational studies, or comparative studies; and were published between 2014 and 2026 in peer-reviewed journals and available on the searched databases.

Exclusion Criteria

Excluded studies were those that: excluded a young female population or lacked sex-disaggregated data; focused solely on surgical interventions without a physiotherapy component; included individuals with acute conditions (acute lateral ankle sprain within six weeks or post-operative ankle repair within three months), subacute conditions (incompletely rehabilitated ankle sprain between six to 12 months or recurrent sprain without confirmed chronicity), fractures, or neurological conditions; were case reports, editorials, letters, narrative reviews, or conference abstracts; or were not retrievable in full text or lacked open access.

Quality Assessment

**Figure 2:**
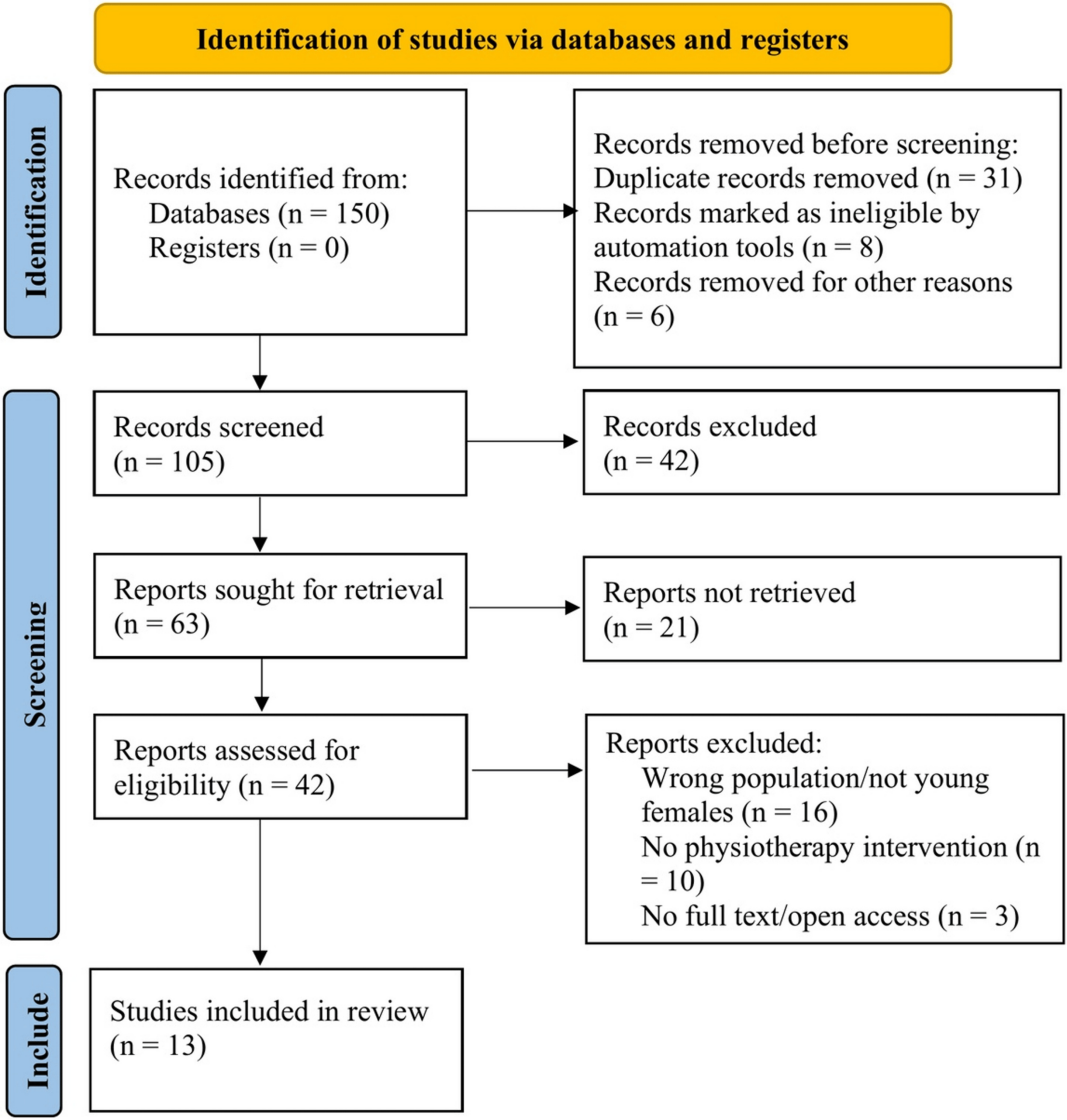
PRISMA flow chart PRISMA: Preferred Reporting Items for Systematic Reviews and Meta-Analyses

To account for heterogeneity across included studies, methodological quality was evaluated using proven methodologies relevant to study designs. An adapted RoB-2 checklist covering randomization, allocation concealment, baseline comparability, assessor blinding, outcome data completeness, intention-to-treat analysis, and between-group statistical comparisons was used to evaluate RCTs [9]. Cross-sectional studies were appraised using the 20-item AXIS (appraisal tool for cross-sectional studies) checklist [10]. Quality evaluations were conducted separately by two reviewers; discrepancies were resolved through discussion. The article selection procedure is shown in the PRISMA flow diagram (Figure 2). 

Results

Thirteen published articles in all fulfilled the predetermined qualifying requirements for this study, offering a thorough summary of evidence-based physiotherapy treatments for young female populations with persistent ankle instability.

The 13 included studies comprised eight RCTs and five cross-sectional observational and correlational studies. Sample sizes ranged from 30 to 258 participants, with a collective total of 787 participants across all included studies. Intervention durations among RCTs varied from four to 12 weeks. The details of the included studies are summarized in Table 1.

**Table 1:** Summary of evidence-based physiotherapy interventions for CAI in young females CAI: chronic ankle instability; RCT: randomized controlled trial; FAAM: Foot and Ankle Ability Measure; FAAM-S: Foot and Ankle Ability Measure-Sports Subscale; CAIT: Cumberland Ankle Instability Tool; FAI: functional ankle instability; SEBT: Star Excursion Balance Test; YBT: Y-Balance Test; DNS: dynamic neuromuscular stabilization; WBV: whole-body vibration; ROM: range of motion; HRQOL: health-related quality of life; PFP: patellofemoral pain syndrome; DKV: dynamic knee valgus; ASI: asymmetry index

Study Title	Year	Author	Study Design	Age	Sample Size	Results	Remark
Rehabilitation for chronic ankle instability with or without destabilization devices: a randomized controlled trial [12]	2016	Donovan et al.	RCT	18-25 years	n = 41 (55% female)	4-week rehabilitation with destabilization devices improved FAAM, ROM, strength, and balance in young adults with CAI. Both groups improved; the device group showed greater gains in self-reported function. Female participants showed comparable outcomes to males.	Supports the use of functional destabilization devices as an adjunct in CAI rehabilitation. Female-dominant sample strengthens applicability to the young female population.
Effects of whole-body vibration and balance training on female athletes with chronic ankle instability [13]	2021	Chang et al.	RCT	18-25 years	n = 63 (all female athletes)	In female athletes with CAI, 6-week WBV and balance training enhanced SEBT, joint position sensing, and isokinetic strength. Anteromedial, posterolateral, and lateral reach directions were all significantly improved in the WBV group (p<0.05). The two active groups did better than the controls.	All-female sample directly relevant to this review. WBV is a viable adjunct intervention for improving dynamic strength and balance in young female athletes with CAI.
Comparative effects of neuromuscular- and strength-training protocols on pathomechanical, sensory-perceptual, and motor-behavioral impairments in patients with chronic ankle instability: randomized controlled trial [14]	2022	Kim et al.	RCT	18-40 years	n = 67 athletes (3 groups)	Neuromuscular training demonstrated superior improvements in pathomechanical, sensory-perceptual, and motor-behavioral outcomes compared to strength training alone. Both protocols yielded significant within-group improvements.	Highlights multidimensional benefits of neuromuscular training over isolated strength training in CAI; supports inclusion of sensory-perceptual components in evidence-based rehabilitation protocols.
Comprehensive corrective exercise program improves ankle function in female athletes with limited weight-bearing ankle dorsiflexion: a randomized controlled trial [15]	2024	Sohrabi et al.	RCT	18-30 years	n = 30 female athletes	A comprehensive corrective exercise program significantly improved ankle function and weight-bearing dorsiflexion ROM in female athletes. The intervention group demonstrated greater functional gains compared to the control.	All-female sample directly relevant to this review. Supports targeted corrective exercise addressing dorsiflexion deficits as a key component of CAI rehabilitation in young female athletes.
Comparison of the effects of exergaming and balance training on dynamic postural stability during jump-landing in recreational athletes with chronic ankle instability [16]	2024	Sepasgozar Sarkhosh et al.	RCT	18-35 years	n = 34 (CAI)	Both exergaming and balance training significantly improved dynamic postural stability during jump-landing tasks. Exergaming produced comparable outcomes to conventional balance training, with potential advantages in patient engagement and motivation.	Supports exergaming as an evidence-based, technology-augmented alternative to conventional balance training for CAI. Findings applicable to young active females engaged in recreational sport.
Prevalence and impact of chronic ankle instability in female sport: a cross-sectional study [17]	2025	Forsyth L, Donovan L, Martin-Smith R, Rowe PL	Cross-sectional observational (online survey)	16-40 years	n = 258 females	CAI is prevalent across all four sports. Females with CAI reported reduced ankle function (FAAM-S, p<0.05) and lower quality of life (HRQOL) vs. copers and uninjured controls. Women are more prone to report ankle instability than functional deficit. No significant sport-specific differences.	Highlights underreporting of CAI in female sport; supports the need for globally implemented targeted rehabilitation and prevention strategies.
Effect of combined balance exercises and kinesio taping on balance, postural stability, and severity of ankle instability in female athletes with functional ankle instability [18]	2022	Mahmoudzadeh Khalili S, Barati AH, Oliveira R, Nobari H	RCT	18-30 years	n = 30 female athletes with FAI (experimental and control groups)	Combined balance exercises with kinesio taping significantly improved balance, postural stability, and reduced the severity of ankle instability compared to balance exercises alone.	First study to combine kinesio taping with balance exercises specifically in female athletes with FAI; limited to the female population; short-term outcomes only.
Dynamic neuromuscular stabilization, balance, and conventional training for chronic ankle instability in amateur athletes: a randomised controlled trial [19]	2025	Yesilkir S, Ergezen Sahin G	RCT	18-25 years	n = 45 amateur athletes with CAI (3 groups: DNS, balance training, conventional training)	Dynamic Neuromuscular Stabilization (DNS) training showed superior improvements in balance and functional outcomes compared to balance and conventional training groups.	Highlights DNS as a promising intervention; conducted in amateur athletes, limiting generalizability to professional/elite populations.
Spatiotemporal gait characteristics and ankle kinematics of backward walking in people with chronic ankle instability [20]	2020	Balasukumaran T, Gottlieb U, Springer S	Cross-Sectional Observational Study	Not reported	n = 30 (15 CAI, 15 healthy controls)	People with CAI showed modified spatiotemporal gait parameters and ankle kinematics during backward walking, with reduced velocity, stride length, and altered ankle motion compared to controls.	Novel use of backward walking as an assessment tool for CAI; small sample size; cross-sectional design limits causal inference.
Adding neurofeedback training to neuromuscular training for rehabilitation of chronic ankle instability: a 3-arm randomized controlled trial [21]	2024	Yalfani A, Azizian M, Gholami-Borujeni B	RCT	18-25 years	n = 62 (young adults with CAI)	Adding neurofeedback to neuromuscular training produced superior improvements in balance and functional outcomes compared to neuromuscular training alone. All active groups outperformed controls.	Introduces neurofeedback as an adjunct neuromodulatory intervention in CAI rehabilitation; supports multimodal training approaches combining sensory feedback with neuromuscular protocols.
Comparing kinematic asymmetry and lateral step-down test scores in healthy, CAI, and patellofemoral pain syndrome female basketball players: a cross-sectional study [22]	2023	Emamvirdi M, Hosseinzadeh M, Letafatkar A, Thomas AC, Dos'Santos T, Smania N, et al.	Cross-sectional observational (between-subject)	18-30 years	n = 60 female basketball players: CAI (n=20), PFP (n=20), healthy controls (n=20)	Ankle dorsiflexion ROM is significantly less in CAI and PFP vs. healthy. Dynamic knee valgus is greater in CAI and PFP bilaterally (p<0.001). Lateral step-down scores worse in CAI and PFP vs. healthy (p=0.001). Greatest asymmetry in DKV angle (ASI, p<0.001).	Underscores the need for physiotherapy addressing dorsiflexion ROM and frontal-plane knee control in female athletes with CAI. Informs rehabilitation screening.
Correlations of strength, proprioception, and dynamic balance to the Cumberland Ankle Instability Tool Score among patients with chronic ankle instability: a cross-sectional study [23]	2024	Peng D, Tang H, Mao M, Song Q, Mao D, Wang J, Sun W	Cross-sectional correlational	Mean 22.47 ± 2.36 years	n = 34 (CAI)	Plantarflexion and inversion muscle strength positively correlated with CAIT scores. Inversion proprioception negatively correlated with CAIT (p<0.05). SEBT posteromedial direction significantly correlated with CAIT (r=0.680, p=0.001). Anterior and posterolateral SEBT directions are not significant.	Supports targeted neuromuscular rehabilitation focusing on plantarflexion/inversion strength, inversion proprioception, and posteromedial balance to improve subjective stability in CAI.
Comparison of functional movement, balance, vertical jumping, hip strength and injury risk in adolescent female volleyball players with and without chronic ankle instability [24]	2025	Akoğlu AS, Adin RM, Ada AM, Bayrakci Tunay V, Erden Z	Cross-Sectional Comparative Study	14-18 years	n = 60 adolescent female volleyball players (30 CAI, 30 without CAI)	Volleyball players with CAI demonstrated significantly poorer functional movement screen scores, balance, vertical jump performance, and hip strength, along with higher injury risk compared to those without CAI.	Highlights multi-domain deficits in CAI beyond the ankle joint, specific to adolescent female volleyball players; cross-sectional design precludes causality.

Assessment of Risk of Bias

The methodological quality of included studies varied by design. A common limitation across the RCTs was the lack of blinding for both participants and therapists, which is a characteristic of therapeutic exercise studies. However, all RCTs employed blinded assessors for outcome measurement. All RCTs clearly reported randomization procedures and demonstrated baseline comparability between groups. The reviewers' inter-rater agreement was high (k = 0.84). Eight RCTs were included, and risk was assessed using the RoB-2 tool (Table 2).

**Table 2:** Quality assessment of randomized controlled trials using Risk of Bias-2 (RoB-2)

Study	Domain 1	Domain 2	Domain 3	Domain 4	Domain 5	
	Bias Arising from the Randomisation Process	Bias Due to Deviations from Intended Interventions	Bias Due to Missing Outcome Data	Bias in Measurement of the Outcome	Bias in Selection of the Reported Result	Overall Risk of Bias
Donovan et al. [12]	Low	Some Concerns	Low	Some Concerns	Some Concerns	Some Concerns
Chang et al. [13]	Low	Low	Low	Low	Low	Low
Kim et al. [14]	Low	Low	Low	Low	Low	Low
Sohrabi et al. [15]	Low	Low	Low	Low	Low	Low
Sepasgozar Sarkhosh et al. [16]	Low	Low	Low	Low	Low	Low
Mahmoudzadeh Khalili et al. [18]	Low	Some Concerns	Low	Low	Low	Some Concerns
Yesilkir & Ergezen Sahin [19]	Low	Low	Low	Low	Low	Low
Yalfani et al. [21]	Low	Low	Low	Low	Low	Low

Assessment Tool

Every study outlined its objectives in detail and used appropriate designs. A common limitation was the lack of formal sample size justification and possible bias in selection because of convenience or volunteer sampling. Among the cross-sectional studies appraised using the AXIS tool, all five studies clearly stated their aims, employed appropriate study designs, adequately described their basic data, and reported internally consistent results with justified conclusions. Nonetheless, significant methodological flaws were found in every study. With the exception of Balasukumaran et al., four of the five studies lacked sample size justification. The selection process was rated as non-representative across all five studies, and none of the studies provided information regarding non-responders or addressed concerns related to non-response bias. The sampling frame was considered appropriate in Forsyth et al., Peng et al., and Akoğlu et al., but not in Balasukumaran et al. or Emamvirdi et al. [17,20,22-24]. Notably, only Forsyth et al. reported measures taken to address non-responders. Despite these limitations, all five studies demonstrated strengths in outcome measurement, use of previously piloted or published tools, statistical reporting, and adherence to ethical norms, such as disclosure of funding or conflicts of interest and informed consent. The five cross-sectional studies were evaluated by the AXIS tool (Table 3).

**Table 3:** Quality assessment of included cross-sectional studies using the AXIS tool AXIS tool: appraisal tool for cross-sectional studies

AXIS appraisal question	Forsyth et al. [17]	Balasukumaran et al. [20]	Emamvirdi et al. [22]	Peng et al. [23]	Akoğlu et al. [24]
Were the aims/objectives of the study clear?	Yes	Yes	Yes	Yes	Yes
Was the study design appropriate for the aims?	Yes	Yes	Yes	Yes	Yes
Was the sample size justified?	No	Yes	No	No	No
Was the target population clearly defined?	Yes	Yes	Yes	Yes	Yes
Was the sampling frame appropriate?	Yes	No	No	Yes	Yes
Was the selection process representative?	No	No	No	No	No
Were measures taken to address non-responders?	Yes	No	No	No	No
Were the outcome variables measured appropriately?	Yes	Yes	Yes	Yes	Yes
Were the tools piloted/published previously?	Yes	Yes	Yes	Yes	Yes
Was statistical significance clearly determined?	Yes	Yes	Yes	Yes	Yes
Were the methods described in enough detail?	Yes	Yes	Yes	Yes	Yes
Were the basic data adequately described?	Yes	Yes	Yes	Yes	Yes
Is there concern regarding non-response bias?	No	No	No	No	No
Was the info about non-respondents described?	No	No	No	No	No
Were the results internally consistent?	Yes	Yes	Yes	Yes	Yes
Were all method-defined results presented?	Yes	Yes	Yes	Yes	Yes
Were the discussions and conclusions justified?	Yes	Yes	Yes	Yes	Yes
Were the study limitations discussed?	Yes	Yes	Yes	Yes	Yes
Were funding/conflicts of interest reported?	Yes	Yes	Yes	Yes	Yes
Was ethical approval/informed consent attained?	Yes	Yes	Yes	Yes	Yes

Discussion

The current systematic review summarizes the available data from 13 studies and demonstrates that structured, multimodal physiotherapy is highly effective in improving neuromuscular control, dynamic balance, proprioception, and functional performance in young females with CAI. No single intervention modality consistently outperformed all others in efficacy, according to indirect comparisons across included studies rather than head-to-head trials; instead, the biggest improvements were seen when several targeted approaches were combined within a progressive rehabilitation framework [12,13,19].

Balance and neuromuscular training formed the cornerstone intervention across the majority of included studies. Chang et al. demonstrated the superior effectiveness of whole-body vibration combined with balance training in an exclusively female athlete cohort, with significant improvements across SEBT, joint position sense, and isokinetic strength [13]. This finding is consistent with the broader WBV literature, suggesting that vibration-induced activation of muscle spindles and Ia afferents augments proprioceptive feedback, thereby addressing a core mechanism underlying functional instability in young women. Similar to this, Yesilkir and Ergezen Sahin found that while dynamic neuromuscular stabilization (DNS) training, which incorporates breathing, postural control, and movement patterning from a developmental neurology framework, produced large effect sizes (η² = 0.92-0.97), they should be interpreted cautiously because there was no participant blinding and the sample sizes were small (n=45 across three groups), both of which are known causes of effect size inflation in exercise intervention trials [19].

Complementing these findings, Mahmoudzadeh Khalili et al. demonstrated that combining balance exercises with kinesio taping produced significant improvements in balance, postural stability, and self-reported severity of ankle instability in female athletes with functional ankle instability [18]. Peng et al. identified significant correlations between strength, proprioception, and dynamic balance with Cumberland Ankle Instability Tool scores, highlighting the interdependent relationship between sensorimotor deficits and perceived ankle instability in individuals with CAI [23].

The role of proximal and core muscle function in CAI management emerged as a clinically important theme. Forsyth et al. reported a high prevalence and substantial functional impact of CAI in female sport populations, reinforcing the broader clinical burden of CAI in young female athletes [17]. All of these results point to the possibility that rehabilitation for young females with CAI should routinely incorporate proximal stabilization exercises targeting the trunk and hip musculature alongside traditional ankle-focused interventions.

Kim et al. suggested that neuromuscular training protocols demonstrated superior improvements in pathomechanical and sensory-perceptual deficits compared to strength training alone in patients with CAI, reinforcing the multidimensional rehabilitation approach advocated in evidence-based physical therapy management [14]. A comprehensive corrective exercise program done by Sohrabi et al. focuses on targeting limited weight-bearing ankle dorsiflexion and significantly improved ankle function in female athletes, directly supporting the inclusion of mobility-focused interventions within evidence-based physical therapy protocols for young females with CAI [15].

The cross-sectional comparative study by Akoğlu et al. corroborated this proximal deficit model by demonstrating that adolescent female volleyball players with CAI exhibited significantly poorer functional movement screen scores, reduced vertical jump performance, and diminished hip strength compared to unaffected peers, alongside elevated injury risk profiles [24]. These multi-domain deficits underscore the systemic functional consequences of CAI and reinforce the rationale for incorporating hip and lower-extremity strengthening within rehabilitation programs targeting young female athletes.

Joint mobilization and manual therapy techniques demonstrated significant efficacy for restoring mechanical ankle function. Dorsiflexion restriction, commonly occurring as a consequence of posterior talar displacement following lateral ankle sprain, is a well-established predictor of CAI recurrence, making restoration of this movement a priority intervention target in young female populations.

Additionally, Wittouck et al. highlighted that anatomical variations in the morphology of the incisura fibularis may contribute to susceptibility for ligamentous ankle injuries, emphasizing that both structural and neuromuscular factors should be considered in the assessment and long-term management of CAI [25].

The gait analysis study by Balasukumaran et al. provided novel mechanistic insight by revealing that individuals with CAI exhibit altered spatiotemporal gait parameters and ankle kinematics during backward walking, including reduced velocity and stride length, compared to healthy controls [20]. Backward walking assessment represents an emerging clinical tool that may sensitively detect residual neuromuscular deficits not apparent during conventional forward-gait analysis, and its findings reinforce the need for comprehensive movement assessment in CAI rehabilitation.

This finding implicates autonomic nervous system regulation and diaphragmatic breathing in the modulation of postural control and ankle stability, opening a novel avenue for adjunct respiratory-based interventions in CAI management that warrants investigation in larger, adequately powered trials.

Wikstrom and Brown emphasized the importance of standardized reporting criteria and the identification of “copers” in CAI research, noting that heterogeneity in participant classification can substantially influence interpretation of rehabilitation outcomes and inter-study comparisons [26]. This methodological consideration is particularly important for future physiotherapy trials investigating intervention efficacy in young female populations with CAI.

Clinical Implications

The results of this review indicate that physical therapy management for CAI should be centered on progressive, task-specific rehabilitation rather than isolated symptom relief. In young females, these interventions may be particularly important because recurrent instability can affect participation confidence, mobility, and long-term musculoskeletal health. Clinicians should also consider patient adherence, exercise supervision, and progression criteria to ensure effective recovery. Overall, the evidence supports an active, impairment-based rehabilitation model as the cornerstone of management for CAI.

In addition to exercise therapy, manual therapy may serve as a useful adjunct when patients present with restricted ankle dorsiflexion, joint hypomobility, or movement limitations that contribute to instability. Mobilization techniques combined with therapeutic exercise can help improve range of motion, optimize joint mechanics, and support more efficient functional movement patterns. The review further suggests that long-term management should emphasize neuromuscular retraining and return-to-activity preparation to reduce recurrence and persistent disability. This evidence may guide clinicians in selecting interventions that are both practical and aligned with current best practice.

CAI tends to affect females more frequently due to a combination of anatomical, hormonal, and biomechanical factors. A wider pelvis increases the Q-angle and shifts mechanical load onto the lateral ankle, while hormones like estrogen reduce ligament stiffness that will cause laxity, making the ankle more vulnerable to repeated giving way. Women also tend to show delayed peroneal muscle activation and land with greater ankle inversion during dynamic tasks, leaving the joint poorly protected at critical moments. Given these sex-specific vulnerabilities, this systematic review was undertaken to identify physiotherapy interventions that most effectively address the root causes of instability in young females, rather than simply managing symptoms. The findings highlight that early, structured rehabilitation combining neuromuscular retraining, balance progression, and proximal strengthening can support long-term joint health in young females.

Limitations

This review has a few limitations. First, the heterogeneity of included study designs, outcome measures, and intervention protocols precluded formal meta-analysis, limiting the ability to quantitatively synthesize effect sizes. Second, although all included studies involved young female participants, the degree to which findings are exclusively attributable to this demographic varies; some studies included male participants alongside females without fully disaggregating outcomes by sex. Third, the small sample sizes characteristic of several included studies limit statistical power and generalizability. Fourth, the restriction to English-language publications in accessible databases may have introduced publication bias. Finally, the 2014-2026 search window, while contemporary, may exclude foundational studies that continue to inform current practice.

Future Scope

Future research should prioritize large-scale, multicenter RCTs with exclusively female cohorts to establish sex-specific evidence-based protocols for CAI rehabilitation. Longitudinal studies examining recurrence rates and long-term functional outcomes beyond 12 months are critically needed. Dose-response investigations examining optimal training volume, frequency, and progression for balance and neuromuscular interventions would substantially advance clinical protocol design. The integration of wearable sensor technology and telerehabilitation platforms is particularly relevant for young active females and warrants investigation. Additionally, qualitative research exploring psychosocial barriers to rehabilitation adherence in young female patients would complement the predominantly quantitative evidence base.

## Conclusions

This systematic review concludes that evidence-based physiotherapy, encompassing progressive balance training, neuromuscular re-education, peroneal and core strengthening, joint mobilization, PNF techniques, and multimodal rehabilitation, is highly effective for managing CAI in young females. Whole-body vibration combined with balance training and dynamic neuromuscular stabilization protocols demonstrated particularly strong evidence, with the included studies reporting moderate to large effect sizes across functional and neuromuscular outcomes. The consistent finding across RCTs and observational studies is that early, structured, and progressive rehabilitation tailored to the specific biomechanical and neuromuscular vulnerabilities of young females optimizes functional recovery, reduces reinjury risk, and improves long-term quality of life. The development of standardized, sex-specific rehabilitation protocols informed by rigorous large-scale RCTs remains the most critical priority for advancing clinical management of this prevalent condition.
